# Atypical hypnotic compound ML297 restores sleep architecture immediately following emotionally valenced learning, to promote memory consolidation and hippocampal network activation during recall

**DOI:** 10.1093/sleep/zsac301

**Published:** 2022-12-13

**Authors:** Jessy D Martinez, William P Brancaleone, Kathryn G Peterson, Lydia G Wilson, Sara J Aton

**Affiliations:** Department of Molecular, Cellular, and Developmental Biology, University of Michigan, Ann Arbor, MI 48109, USA; Undergraduate Program in Neuroscience, University of Michigan, Ann Arbor, MI 48109, USA; Undergraduate Program in Neuroscience, University of Michigan, Ann Arbor, MI 48109, USA; Department of Molecular, Cellular, and Developmental Biology, University of Michigan, Ann Arbor, MI 48109, USA; Department of Molecular, Cellular, and Developmental Biology, University of Michigan, Ann Arbor, MI 48109, USA

**Keywords:** memory consolidation, REM, spindles, hypnotic drug, hippocampus

## Abstract

Sleep plays a critical role in consolidating many forms of hippocampus-dependent memory. While various classes of hypnotic drugs have been developed in recent years, it remains unknown whether, or how, some of them affect sleep-dependent memory consolidation mechanisms. We find that ML297, a recently developed candidate hypnotic agent targeting a new mechanism (activating GIRK1/2-subunit containing G-protein coupled inwardly rectifying potassium [GIRK] channels), alters sleep architecture in mice over the first 6 hr following a single-trial learning event. Following contextual fear conditioning (CFC), ML297 reversed post-CFC reductions in NREM sleep spindle power and REM sleep amounts and architecture, renormalizing sleep features to what was observed at baseline, prior to CFC. Renormalization of post-CFC REM sleep latency, REM sleep amounts, and NREM spindle power were all associated with improved contextual fear memory (CFM) consolidation. We find that improvements in CFM consolidation due to ML297 are sleep-dependent, and are associated with increased numbers of highly activated dentate gyrus (DG), CA1, and CA3 neurons during CFM recall. Together our findings suggest that GIRK1/2 channel activation restores normal sleep architecture— including REM sleep, which is normally suppressed following CFC—and increases the number of hippocampal neurons incorporated into the CFM engram during memory consolidation.

## Graphical Abstract

**Graphical Abstract:**
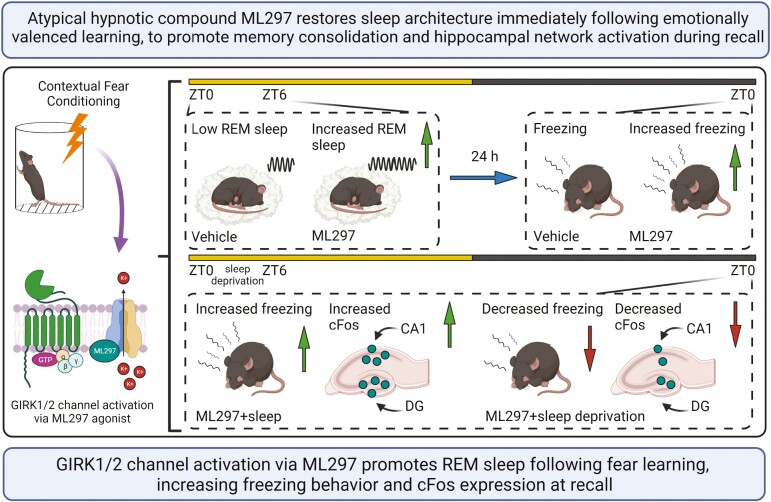

**Statement of Significance**

Both REM and NREM sleep are thought to be important for consolidating hippocampus-dependent memories. We find that GIRK1/2 activator ML297, administered after single-trial fear learning, restores REM sleep that is normally suppressed after learning fearful associations. This restoration is associated with improvements in fear memory storage, resulting in more robust hippocampus activation in the context of subsequent memory recall. Thus, this drug, which also has antiepileptic and anxiolytic properties, may be useful for promoting normal, restorative sleep that benefits memory storage.

## Introduction

Sleep plays an essential role in memory consolidation [1–4]. Available data from both human participants and animal models have implicated both non-rapid eye movement (NREM) and REM sleep in the process of memory storage. While the underlying mechanisms are still under investigation, these states differ from one another, and from the wake, with respect to neuromodulation, neural network oscillatory behavior, neuronal firing patterns, gene expression, and protein translation [3–13].

Sleep loss over the first few hours following training on many hippocampus-dependent tasks leads to long-term memory disruption [1, 14, 15]. Among these, one of the most well-studied is contextual fear memory (CFM), which is initiated by single-trial contextual fear conditioning (CFC) in mice, and is consolidated in a sleep-dependent manner over the next few hours [6, 11, 16–18]. During these first few hours following CFC, both NREM and REM sleep are altered [18–22]. Some of these changes, including enhancements in REM theta (4–12 Hz), NREM spindle (7–15 Hz), and NREM sharp wave-ripple oscillations, predict successful CFM consolidation and recall [18–22]. Both CFC and tone-cued fear conditioning also affect sleep architecture in mice, including transiently suppressing REM sleep [23, 24]. How REM suppression affects CFM consolidation remains unknown. However, data from analogous studies with human participants have suggested that post-conditioning REM sleep time, and limbic system brain activation during REM, predict successful fear memory consolidation [25–27]. Moreover, in mice, theta oscillations present in the dorsal hippocampus during post-CFC REM sleep have been shown to play a causal role in promoting CFM consolidation [18, 28]. Optogenetically driven hippocampal theta activity can even rescue CFM consolidation from the deleterious effects of post-CFC sleep deprivation (SD) [18]. Thus taken together, available data suggest that limbic system activity and oscillations associated with both NREM and REM sleep contribute to the long-term storage of recently encoded fear memories.

Hypnotic drug interventions have recently been used as an experimental strategy to test the relationships between sleep, memory consolidation, and synaptic plasticity [29–35]. The majority of the hypnotics used in these studies—including benzodiazepines, nonbenzodiazepine “z-drugs,” and sodium oxybate—act as positive allosteric modulators of GABA_A_ receptors or as GABA_B_ receptor agonists. These drugs, while effective at promoting NREM sleep, can have unwanted side effects, including over-sedation, electroencephalogram (EEG) anomalies including aberrant oscillations, and memory deficits [29, 30, 34, 36–39]. Recent work has aimed to develop new classes of hypnotic drugs, including orexin receptor antagonists, melatonin receptor agonists, and most recently, activators of G-protein inward rectifying potassium (GIRK) channels [40–42]. GIRK channels consist of four subunits (1–4 or K_ir_3.1–3.4), with homo- or hetero-tetrameric compositions that are specific to organs, brain regions, and cell types (e.g. GIRK1/2 channels are selectively expressed in hippocampal neurons, GIRK1/4 channels are present in cardiac myocytes, and GIRK2/3 channels are present in the midbrain) [43–48]. GIRK channel activation is typically associated with G_i_-mediated intracellular signaling and itself causes neuronal hyperpolarization, leading to reduced neuronal activity [49, 50]. Recent studies have found that the GIRK1/2 subunits can be directly activated independently of G_i_ using a selective and potent compound known as ML297 [51, 52]. Behavioral studies using ML297 in rodents have shown it suppresses seizures, reduces anxiety-like behaviors, and promotes NREM sleep during the circadian active phase (i.e. dark phase) [40, 52, 53]. However, it remains unclear whether, and how, ML297 affects sleep-dependent memory processing.

To characterize the effects of GIRK1/2 channel activation on post-learning sleep and sleep-dependent memory consolidation, we administered ML297 immediately following CFC and measured changes in post-conditioning sleep architecture. We found that while post-CFC NREM sleep was unchanged, ML297 administration restored REM sleep in the hours following CFC (renormalizing it to levels seen at baseline), and significantly improved CFM consolidation. This effect was sleep-dependent—i.e. ML297 had no beneficial effect on CFM when administered during post-CFC SD. Finally, we found that post-CFC sleep, and particularly ML297-augmented post-CFC sleep, led to increased hippocampal cFos and Arc expression during CFM recall. Taken together, our data demonstrate that post-CFC REM sleep plays a critical role in CFM consolidation, leading to greater hippocampal activation during a recall—and that restoration of normal post-CFC REM sleep by ML297 promotes this process.

## Methods

### Animal handling and husbandry

All mouse husbandry, experimental, and surgical procedures were reviewed and approved by the University of Michigan Internal Animal Care and Use Committee. For all experiments, 4–5-month-old, male C57BL/6J mice (Stock No. 000664, Jackson Labs) were housed under a 12:12hr light/dark cycle (lights on at 9 AM) and had *ad lib* access to food and water. Mice were housed with littermates until either EEG implantation surgery or (for non-implanted mice) daily habituation prior to behavioral procedures, at which point they were single housed in standard cages with beneficial environmental enrichment.

### Experimental design and statistical analyses

Male littermates were randomly assigned to treatment groups (*n* = 5–7 per group) at the time of single housing for EEG implantation or behavioral procedures. Data analyses were carried out in a blinded manner; in some cases (e.g. for EEG recordings), data were consensus scored by two individuals to reduce variability. Statistical analyses were carried out using GraphPad Prism software (Version 9.1). For each specific data set, the statistical tests and *P*-values used are listed in the “Results” section and in corresponding figures and figure legends.

### Surgical procedures and EEG recording

For EEG experiments, mice underwent surgical procedures for implantation of electroencephalogram (EEG) and electromyogram (EMG) electrodes. Briefly, mice were anesthetized with 1%–2% isoflurane. Stainless steel screw electrodes for EEG recording and referencing were positioned over primary visual cortex (2.9 mm posterior to Bregma, 2.7 mm lateral) bilaterally and cerebellum, respectively, and a braided stainless steel wire EMG electrode was placed in the nuchal muscle. After 11 days of postoperative recovery, each mouse underwent 3 days of habituation to daily handling (5 min/day) and tethering to recording cables in their home cage. Following habituation, 24-hr baseline recordings were made from each mouse, starting at lights-on (ZT0). Subsequently, for studies of sleep-dependent memory consolidation, mice underwent CFC training at lights on (ZT0) the following day, and were recorded for an additional 24 hr thereafter. EEG/EMG signals (0.5–300 Hz) were amplified at 20 ×, digitized, further digitally amplified at 20–100 ×, and continuously recorded (with a 60-Hz notch filter) using Plexon Omniplex software and hardware (Plexon Inc.) as previously described [18, 19, 54, 55].

### Sleep state and power spectra analysis

Baseline and post-CFC recordings were scored in 10-sec epochs as wake, NREM, or REM sleep using custom MATLAB software. EEG and EMG data were band-pass filtered at 0–90 Hz and 150–250 Hz, respectively, for viewing during scoring. Raw EEG data (0.5–300 Hz) were used for fast-Fourier transform and generation of power spectral density from 0.5 to 20 Hz using NeuroExplorer 5 software (Plexon Inc.). An automated spindle detection algorithm was used to identify sleep spindles in band-pass filtered EEG data (7–15 Hz), as intervals containing ≥6 successive deviations (i.e. peaks or troughs) of signal that surpassed mean signal amplitude by 1.5 standard deviations, lasting between 0.25 and 1.75 sec [55].

### CFC, drug administration, sleep monitoring, and sleep deprivation

Mice underwent single-trial CFC as previously described [6, 11, 18–20]. Each mouse was placed in a novel cylindrical conditioning chamber made of clear Plexiglas with a metal grid floor and distal cues (Med Associates). Mice were allowed to freely explore for 2 min and 28 sec, after which they received a 0.75 mA, 2-sec foot shock through the grid floor, followed by an additional 30 sec in the CFC chamber. Immediately following CFC, mice were returned to their home cage and given an i.p. injection of either ML297 (30 mg/kg; Tocris) or vehicle (2% DMSO in 0.5% hydrooxypropyl cellulose aqueous solution). Injections occurred within 5 min of removal from the CFC chamber. CFM tests were conducted 24 hr later by returning mice to the CFC chamber for 5 min. Mice were video monitored continuously during both CFC training and CFM testing, and both freezing behavior and time-in-location (Supplementary Figure S1, S8) within the CFC chamber were quantified in a semi-automated manner using Ethovision XT 16 software (Noldus). Freezing was first scored based on transient periods of immobility, as described previously [56], and was verified offline based on the assessment of characteristic freezing-associated posture [18, 54]. CFM-associated freezing behavior was quantified by subtracting each mouse’s % freezing time during pre-shock baseline from the % freezing time across the entire CFM test, as described previously [11, 18, 19].

Following CFC, mice were either allowed *ad lib* sleep (Sleep) or were sleep-deprived (SD) via gentle handing over the next 6 hr (ZT0-6). This method of SD was chosen based on prior work showing that stress response (e.g. glucocorticoid production) evoked by gentle handling SD is not sufficient to disrupt consolidation of fear memory (and in fact may enhance consolidation) [57–59]. Following SD, all mice were allowed *ad lib* recovery sleep over the next 18 hr prior to CFM testing. For mice without EEG/EMG implants, sleep was quantified over the first 6 hr post-CFC via visual monitoring. Every 5 min, individual mice were scored as awake or asleep, with sleep identification based on immobility, slow breathing, and presence of stereotyped (crouched) sleep postures, consistent with prior studies. For SD, gentle handling procedures included cage tapping or shaking, or nest disturbance (Fisher et al., 2012; Delorme et al., 2019, 2021; Puentes-Mestril et al., 2021). EEG/EMG-based validation of both the visual sleep scoring method, and SD methodology, are shown in Supplementary Figure S7.

### Histology and immunohistochemistry

To quantify hippocampal activation patterns associated with the recall, 90 min following the conclusion of CFM tests, mice were euthanized with an overdose of sodium pentobarbital and perfused with ice-cold PBS, followed by ice-cold 4% paraformaldehyde. Brains were dissected, post-fixed, and cryoprotected in a 30% sucrose solution. 50 µm coronal dorsal hippocampal sections were immunostained using rabbit-anti-cFos (1:1000; Abcam, ab190289) and guinea pig-anti-Arc (1:500; Synaptic Systems, 156004) as markers of neuronal activation. Secondary antibodies used included Alexa Fluor 488 (1:200; Invitrogen, A11032) and Alexa Fluor 594 (1:200; Invitrogen, A11034). Stained sections were mounted using Prolong Gold antifade reagent with DAPI (Invitrogen, P36931) and imaged using a Leica SP8 confocal microscope with a 10X objective, to obtain z-stack images (10 µm steps) for maximum projection of fluorescence signals. Identical image acquisition settings (e.g. exposure times, frame average, and pixel size) were used for all sections.

For analysis of hippocampal activation patterns, three images of dorsal hippocampus were taken per mouse and equally sized regions of interest (ROIs) for DG, CA1, and CA3 regions were obtained for each image. cFos+ and Arc+ neurons were identified and quantified in subregions of these ROIs (i.e. pyramidal or granule cell layers, DG hilus) by a scorer blinded to animal condition, using ImageJ software. For Arc expression in pyramidal cell layers of CA1 and CA3, mean fluorescent intensity (MFI) was measured by adaptive thresholding of fluorescent signals and subtracting the fluorescence intensity of each region from mean background fluorescence [12].

## Results

### GIRK channel activation renormalizes REM sleep architecture in the hours immediately following CFC and improves CFM consolidation

**Figure 1.**
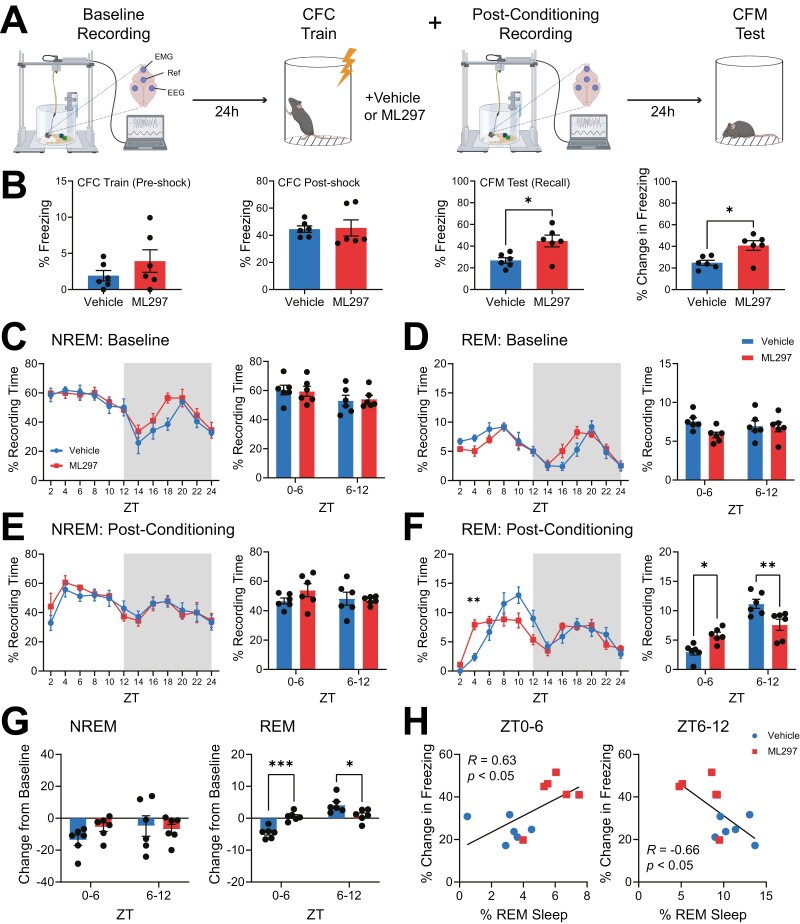
**GIRK1/2 activation restores REM sleep amounts during the first few hours of fear memory consolidation and improves fear memory recall.** (**A**) Configuration of EEG electrodes for sleep recording and schematic of experimental design. Mice were recorded over a 24-hr baseline starting at lights-on (ZT0), then underwent single-trial contextual fear conditioning (CFC) followed by an i.p. injection of vehicle or GIRK1/2 activator ML297 (30 mg/kg). Each mouse was recorded for an additional 24 hr prior to being tested for contextual fear memory (CFM). (**B**) During CFC training mice in the two groups displayed similar freezing behavior. During CFM testing, freezing behavior was significantly greater in ML297-treated mice. Bars indicate mean ± SEM; *n* = 6 mice/group; * indicates *p* = 0.0141 (total freezing during recall), * indicates *p* = 0.0101 (change in freezing from the pre-shock period during CFC), two-tailed, unpaired *t*-test. (***C***) NREM and (***D***) REM sleep behavior during baseline across the light: dark cycle for vehicle- and ML297-treated mice. Shaded areas represent lights off. No changes were seen in time spent in NREM or REM sleep across the light: dark cycle nor in total NREM or REM sleep across 6 hr quartiles. *n* = 6 mice/group. (**E–F**) Sleep behavior post-conditioning across the light: dark cycle for vehicle- and ML297-treated mice. Shaded areas represent lights off. No changes were seen in time spent in NREM across the light: dark cycle. Time spent in REM sleep was significantly altered during the light cycle. REM sleep is significantly increased 3–4 hrs post-treatment of ML297 compared to vehicle controls at this same time point. Values indicate mean ± SEM; *n* = 6 mice/group; ** indicates *p* = 0.0057, Sidak’s *post hoc* test vs. vehicle. ML297 reorganized sleep to show significantly more total REM sleep during ZT0-6 and significantly less total REM sleep during ZT6-12. Sidak’s *post hoc* test vs. vehicle. * indicates *p* = 0.0226 (ZT0-6) and *p* = 0.0045 (ZT6-12), Sidak’s *post hoc* test vs. vehicle. (**G**) Compared to baseline, REM sleep in the first 6 hr post-CFC was more suppressed in vehicle-treated vs. ML297-treated mice, while significantly promoted during the last 6 hr post-CFC. *** and * indicates *p* = 0.0004 (ZT0-6) and *p* = 0.0169 (ZT6-12), respectively, Sidak’s *post hoc* test vs. vehicle. (***H***) Correlation between freezing behavior and % time spent in REM sleep in the first 6 hr post-CFC and last 6 hr post-CFC of the light cycle. *R* and *P* values are shown for Pearson correlation.

**Figure 2.**
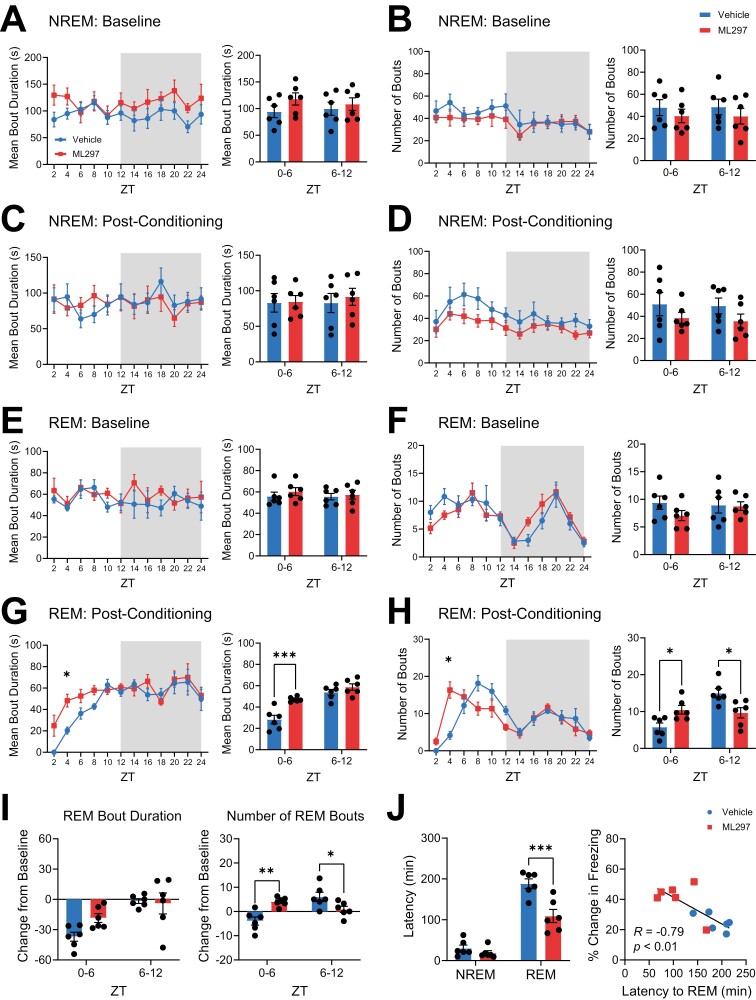
**ML297 REM sleep architecture is promoted during post-conditioning with GIRK channel activation.** (***A***) Mean bout duration and (***B***) number of bouts for NREM sleep over the 24 hr during baseline in the vehicle and ML297 groups were similar. (***C***) Mean bout duration and (***D***) number of bouts for NREM sleep over the 24 hr post-CFC in vehicle- and ML297-treated mice were similar. (***E***) Mean bout duration and (***F***) bout numbers for REM sleep over the 24 hr during baseline in the vehicle and ML297 groups were similar. (***G***) Mean bout duration and (***H***) bout numbers for REM sleep over the 24 hr post-CFC in vehicle- and ML297 treated mice. * indicates *p* = 0.0400 (REM bout duration), *p* = 0.0177 (number of REM bouts), Sidak’s *post hoc* test vs. vehicle. Values indicate mean ± SEM. (***G***) REM mean bout durations and (***H***) bout numbers are shown for hours 0–6 and 6–12 post-CFC. ML297-treated mice show longer REM bout durations during ZT0-6. ** indicates *p* = 0.0003, Sidak’s *post hoc* test vs. vehicle. ML297-treated mice show a greater number of REM sleep bouts during ZT0-6 and a reduced number of REM sleep bouts during ZT6-12. * indicates *p* = 0.0267 (ZT0-6), *p* = 0.0123 (ZT6-12), Sidak’s *post hoc* test vs. vehicle. (***I***) Post-CFC changes in REM mean bout duration (*left*) and number (*right*) from time-matched baseline values. ML297 administration led to a relative increase in the number of REM sleep bouts in the first 6 hr post-CFC, and a decrease in bouts in the last half of the light cycle. ** indicates *p* = 0.0019 (ZT0-6) and * indicates *p* = 0.0419 (ZT6-12), Sidak’s *post hoc* test vs. vehicle. (***J***) ML297-treated mice showed decreased latency to REM sleep (but unchanged latency to NREM sleep) after CFC (*** indicates *p* = 0.0001, Sidak’s *post hoc* test vs. vehicle). Shorter REM latency predicted successful CFM consolidation. *R* and *P* values are shown for Pearson correlation. *n* = 6 mice/group.

To test how GIRK1/2 channel activation affects post-CFC sleep and sleep-dependent CFM consolidation, we recorded ML297-induced changes in sleep architecture and EEG activity following CFC (Figure 1–2). 4–5 month old, male C57BL/6J mice underwent continuous 24-hr baseline EEG/EMG recording starting at lights-on (ZT0), followed by single-trial CFC at ZT0 the following day. Immediately following CFC, mice were administered either vehicle or ML297 (30 mg/kg, i.p.), and underwent EEG recording for an additional 24 hr prior to CFM testing at ZT0 on the third day (Figure 1A). Automated scoring during CFC and CFM testing was used to quantify both CFC- and CFM-associated freezing behavior (Figure 1B). Representative heat maps showing time spent at different locations within the conditioning chamber during training and testing can be seen in Supplementary Figure S1. During initial training (prior to foot shock), vehicle and ML297 groups showed comparable, low levels of freezing behavior (two-tailed, unpaired *t*-test; *p* = 0.2663; t, *df* = 1.177, 10). During the post-shock period of CFC, freezing amounts were likewise comparable between vehicle and ML297 groups (two-tailed, unpaired *t*-test; *p* = 0.8990; *t*, *df* = 0.1419, 10), suggesting similar initial behavioral responses during encoding. However, during CFM testing (i.e. recall), both total freezing (two-tailed, unpaired *t*-test; *p* = 0.0141; t, df = 2.969, 10) and the change in freezing from pre-shock values (two-tailed, unpaired *t*-test; *p* = 0.0101; *t*, *df* = 3.162, 10), were higher in ML297-treated mice than vehicle-treated controls (Figure 1B). This suggests that CFM consolidation was improved by post-CFC administration of ML297.

To test whether enhanced CFM consolidation following ML297 administration correlated with differences in sleep architecture, we first compared baseline vs. post-CFC sleep amounts between vehicle and ML297 groups. Vehicle and ML297 groups showed similar NREM, REM, and wake amounts across the 24-hr baseline recording period (Figure 1C–D, Supplementary Figures S2A–B, S3A, S4A, C), as well as similar NREM sleep, amounts over the next 24 hr post-CFC (two-way repeated measures (RM) ANOVA; *p*(time of day × treatment) = 0.8121; *F*(11, 110 = 0.6157); Figure 1E). Wake amounts post-CFC were similar as well (two-way RM ANOVA; *p*(time of day × treatment) = 0.7915; *F*(1,110) = 0.6394; Supplementary Figure S3B). However, ML297 significantly altered the time course of REM sleep following CFC (two-way RM ANOVA; *p*(time of day × treatment) = <0.0001; *F*(11,110) = 4.209; Figure 1F). This was observed as increased REM sleep amounts over the first 6 hr following CFC in ML297-treated mice (two-way RM ANOVA; *p*(time of day × treatment) = 0.0006; *F*(11,10 = 24.26); Sidak’s *post hoc* test for vehicle versus ML297, *p* = 0.0226; Figure 1F), and reduced REM sleep amount (relative to vehicle-treated controls) over the latter 6 hr of the light phase (ZT6-12; Sidak’s *post hoc* test for vehicle vs. ML297, *p* = 0.0045; Figure 1F). CFC reduced REM sleep during the first 6 hr post-CFC (relative to baseline) in vehicle-treated mice (two-way RM ANOVA; *p*(time of day × treatment) = 0.0003; *F*(1,10 = 30.57)), consistent with previous findings [23, 24]. ML297 reversed this effect over ZT0-6 by increasing REM sleep during this same time period (Sidak’s *post hoc* test for vehicle versus ML297, *p* = 0.0004; Figure 1D, F–G). ML297 did not affect the proportion of time spent in NREM at any timepoint compared with vehicle (two-way RM ANOVA; *p*(time of day × treatment) = 0.1657; *F*(1,10 = 2.236)), the change in NREM amounts from baseline following CFC (two-way RM ANOVA; *p*(time of day × treatment) = 0.0749; *F*(1,10 = 3.953); Figure 1C, E, G), or the proportion of time spent in NREM or REM during the dark phase following CFC (ZT12-24) (Supplementary Figure S2C–D). Critically, freezing behavior at CFM recall was positively correlated with the proportion of time spent in REM sleep over the first 6 hr post-CFC (Pearson correlation coefficient, *R* = 0.6319; *p* = 0.0275), but negatively correlated with REM amounts over the subsequent 6 h (i.e. ZT6-12; Pearson correlation coefficient, *R* = −0.6550, *p* = 0.0208; Figure 1H).

We also quantified how CFC and ML297 affected other features of sleep architecture, including NREM and REM bout durations and bout numbers. These aspects of NREM and REM sleep were similar at baseline between the vehicle and ML297 groups (Figure 2A–B, E–F). Following CFC, both vehicle and ML297 groups had comparable NREM bout duration (two-way RM ANOVA; *p*(time of day × treatment) = 0.4974; *F*(11,110 = 0.9487); Figure 2C) and bout numbers (two-way RM ANOVA; *p*(time of day × treatment) = 0.6252; *F*(11,110 = 0.8149); Figure 2D). In contrast, over the first 6 hr post-CFC, ML297-treated mice showed significantly increased REM sleep bout durations compared to vehicle-treated counterparts (two-way RM ANOVA; *p*(time of day × treatment) = 0.0245; *F*(1,10 = 6.994); Sidak’s *post hoc* test for vehicle vs. ML297, *p* = 0.0003; Figure 2G) and bout numbers (two-way RM ANOVA; *p*(time of day × treatment) = 0.0016; *F*(1,10 = 18.39); Sidak’s *post hoc* test, *p* = 0.0267; Figure 2H). Group differences in wake bout duration or number were not observed, either at baseline or post-CFC (Supplementary Figure S4). In the hours immediately following CFC, vehicle-treated mice showed both reduced REM bout duration and number (relative to baseline); ML297 restored REM bout numbers to baseline levels during this same period (Sidak’s *post hoc* test for vehicle vs. ML297, *p* = 0.0019; Figure 2I). Finally, latency to the first bout of post-CFC REM sleep (but not NREM sleep) was significantly reduced in mice administered ML297 (two-way RM ANOVA; *p*(sleep state × treatment) = 0.0052; *F*(1,10 = 12.67); Sidak’s *post hoc* test for vehicle vs. ML297, *p* = 0.0001; Figure 2J). Reduced latency to REM following CFC also predicted successful CFM recall the following day, with mice with the shortest latency to REM showing the highest levels of freezing (Pearson correlation coefficient, *R* = −0.7924, *p* = 0.0021; Figure 2J).

Taken together, these data suggest that ML297-mediated GIRK channel activation may improve CFM consolidation through a restorative increase in REM sleep over the first few hours following CFC. Thus, ML297 treatment has the effect of renormalizing REM sleep architecture, to offset suppression of REM that typically occurs after CFC and other fear-associated learning [60].

### ML297 administration alters NREM and REM EEG oscillations in a manner consistent with renormalizing sleep architecture

**Figure 3.**
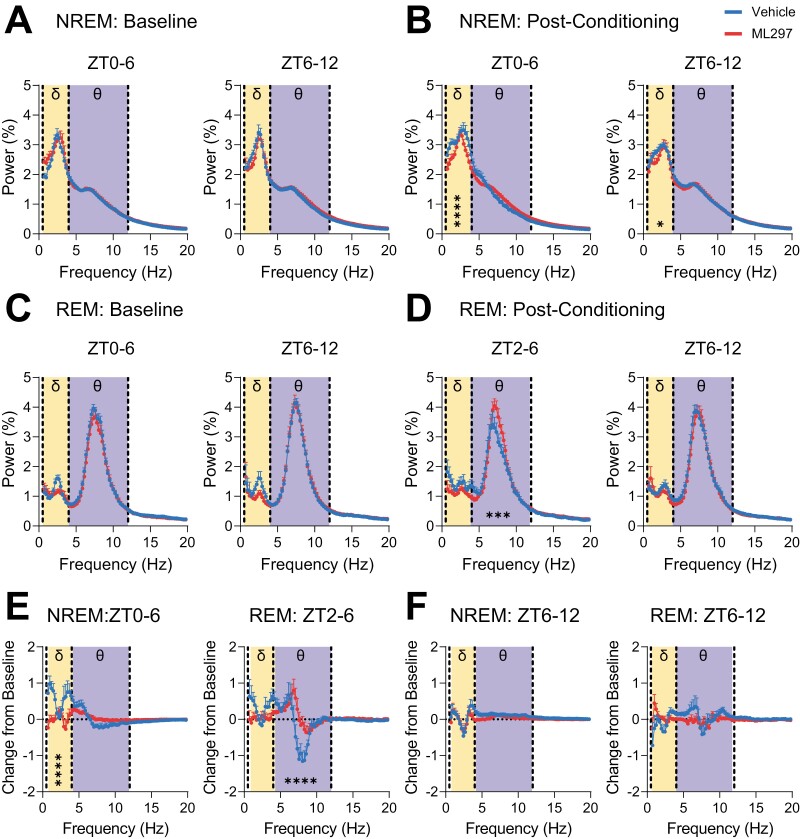
**ML297 has modest effects on overall NREM and REM EEG spectral power.** EEG power spectra (recorded over visual cortex, bilaterally) are shown for vehicle- and ML297-treated mice during NREM baseline (***A***) and post-CFC (***B***), and during REM baseline (***C***) and post-CFC (***D***). Values indicate % of total spectral power at each frequency band, mean ± SEM; *n* = 6 mice/group. For (***B***), **** and * indicate post-CFC differences in NREM delta frequency bands at ZT0-6 and 6-12, respectively, *p* ≤ 0.0001 for 2.9–3.9 Hz, and *p* < 0.05 for 1.4–1.7 Hz, respectively, Sidak’s *post hoc* test vs. vehicle. For (***D***), *** indicates post-CFC differences in REM at ZT2-6, *p* < 0.001 for 7.1–8.1 Hz, Sidak’s *post hoc* test vs. vehicle. Values indicate mean ± SEM; *n* = 6 mice/group. (***E***) Comparisons of changes in spectral power from baseline showed significant differences in NREM delta frequency bands from 0.7–1.9 and 2.9−–3.9 Hz (*p* < 0.0001, Sidak’s *post hoc* test) at ZT0-6 and REM theta frequency bands 6.8–8.3 Hz (*p* < 0.0001, Sidak’s *post hoc* test) at ZT2-6. (***F***) Over the following 6 hr of the light phase (ZT6-12) no changes in spectral power from baseline were observed in either NREM or REM.

We next assessed how NREM- and REM-associated EEG oscillations are affected by ML297 administration. We found that at baseline, vehicle and ML297 groups showed no EEG spectral power differences in either NREM or REM sleep (Figure 3A, C). Following CFC, vehicle- and ML297-treated mice showed NREM spectral power differences (two-way RM ANOVA; *p*(frequency × treatment) <0.0001; *F*(78, 1343 = 3.643)) with vehicle-treated mice having a greater proportion of total EEG power in the NREM delta (0.5–4 Hz) band (Figure 3B). Post-CFC REM EEG spectra also differed between groups, with ML297-treated mice having greater proportional spectral power in the theta (4–12 Hz) band (two-way RM ANOVA; *p*(frequency × treatment) < 0.0001; *F*(78, 869 = 2.349), Sidak’s *post hoc* test for vehicle vs. ML297, *p* = 0.001; Figure 3D). We assessed the change in power from baseline in both vehicle and ML297 groups during post-CFC NREM and REM sleep. We found changes in NREM delta (two-way RM ANOVA; *p*(frequency × treatment) < 0.0001; *F*(78, 1343 = 5.290)) and REM theta (two-way RM ANOVA; *p*(frequency × treatment) < 0.0001; *F*(78, 869 = 4.082)), during ZT0-6 and ZT2-6, respectively (Figure 3E–F). No differences were observed for NREM- or REM-associated EEG oscillations between groups during the dark phase (Supplementary Figure S5).

**Figure 4.**
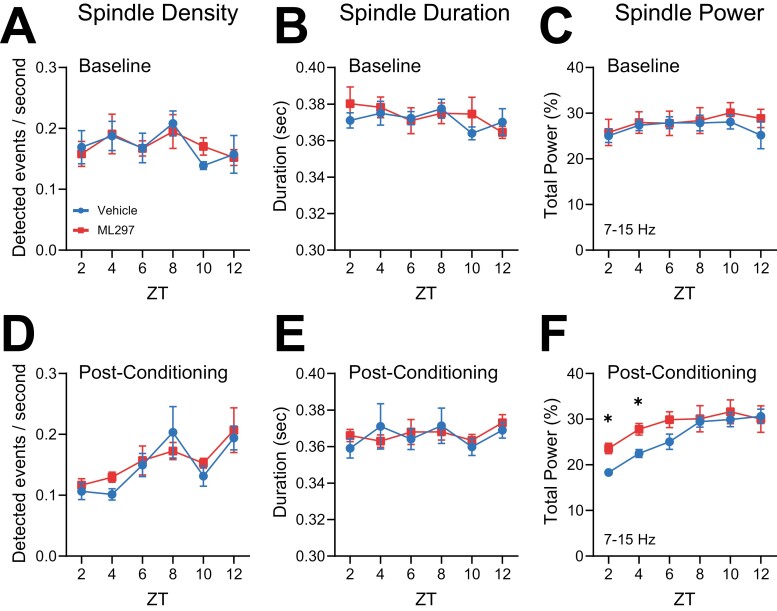
**ML297 normalizes NREM sleep spindle power in the hours following CFC.** NREM spindle density (***A***) and mean duration (***B***) were similar between vehicle and ML297 groups for hours 0–12 during baseline. (***C***) NREM EEG spectral power within the spindle/sigma frequency band (7–15 Hz) was similar between groups at baseline. NREM spindle density (***D***) and mean duration (***E***) were similar between both vehicle- and ML297-treated mice following CFC. (***F***) Over the first 4 hr following CFC, NREM spindle power was higher in ML297-treated mice relative to vehicle-treated mice. * indicates *p* < 0.05, Sidak’s *post hoc* test. Values indicate mean ± SEM; *n* = 6 mice/group.

Because ML297 promoted REM sleep during the first few hours post-CFC, and caused a relative decrease in NREM delta power, we also assessed the effects of both CFC and ML297 on NREM sleep spindles—waxing-and-waning, discrete EEG oscillations with a peak frequency of 7–15 Hz. Spindles are: (1) inversely related to NREM delta power [61–63], (2) implicated in CFM consolidation [21, 64], and (3) critical for transitions between NREM and REM sleep [65, 66]. To test whether alterations in REM sleep architecture after ML297 administration were associated with changes in NREM spindles, we next detected these events in a semi-automated manner [55] and compared post-CFC spindle characteristics between groups. Both at baseline, and following CFC, neither spindle density (two-way RM ANOVA; *p*(time of day × treatment) = 0.7795; *F*(5, 50 = 0.4935)) nor duration (two-way RM ANOVA; *p*(time of day × treatment) = 0.7784; *F*(5, 50 = 0.4951)) differed significantly between ML297—and vehicle-treated mice (Figure 4A–B, D–E). Spindle power during baseline NREM sleep was similar between the two groups and relatively invariant across the entire light phase (ZT0-12; Figure 4C). However, in the first few hours following CFC, the proportion of total NREM EEG spectral power in the spindle (i.e. sigma) band was significantly higher in ML297-treated vs. vehicle-treated mice (two-way RM ANOVA; *p*(time of day × treatment) = 0.0211; *F*(5, 50 = 2.938); Figure 4E). This difference appeared to reflect suppressed spindle power (relative to baseline) in vehicle-treated mice over the first 4 hr of post-CFC NREM sleep, which was reversed by ML297 (Sidak’s *post hoc* test for vehicle vs. ML297 at ZT0-2 and 2–4, *p* = 0.0223 and *p* = 0.0424). This difference in spindle power between the groups following CFC is consistent with both the relative increases in NREM EEG delta power, and the overall suppression of REM, following conditioning in vehicle-treated (but not ML297-treated) mice. Intriguingly, this difference in spindle power continued into the subsequent dark phase (ZT12-24) (two-way RM ANOVA; *p*(time of day × treatment) = 0.0300; *F*(5, 50 = 2.718); Supplementary Figure S6). Together, our EEG data suggest that administration of GIRK channel activator ML297 following CFC modestly augments REM theta, and renormalizes NREM delta/spindle ratios, in a manner consistent with its renormalization of sleep architecture. Because these EEG oscillatory changes are similar to those known to be associated with successful sleep-dependent CFM consolidation [18, 19, 21], they are consistent with state-dependent hippocampal oscillations serving as a potential driver of memory enhancement by ML297 [3, 4].

### ML297 effects on CFM consolidation are sleep-dependent

Because ML297 restores REM sleep architecture and NREM oscillations during a critical time window for CFM consolidation, we next tested whether ML297-mediated improvement in CFM was sleep-dependent, or due to other effects of GIRK activation. In the second cohort of non-instrumented mice, we tested whether post-CFC SD interfered with ML297-driven improvements in CFM consolidation. At lights-on, mice underwent single-trial CFC training and were immediately administered either vehicle or ML297. Over the next 6 hr, mice in each treatment group were either allowed *ad lib* sleep (and visually monitored for changes in sleep amount) or underwent gentle-handling SD in their home cage (which is sufficient to disrupt CFM consolidation) [6, 18]. To further validate this approach, we compared the sleep architecture based on EEG/EMG recordings and visual scoring in the same cohort of mice. We found no difference in total sleep time measured using EEG analysis versus visual observations (**S7A-C**). EEG measurements in SD mice indicated that gentle handling led to 90% of the 6-hr SD period spent in the wake, with minimal NREM bouts and no REM sleep(Supplementary Figure S7D). Over the course of SD, mice underwent a progressively increasing number of experimenter interventions to prevent sleep, as predicted based on accumulating homeostatic sleep drive (Supplementary Figure S7E).

**Figure 5.**
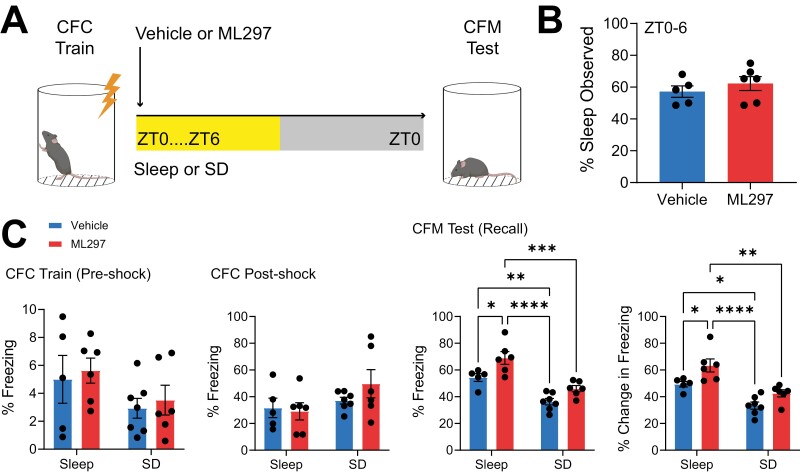
**Post-CFC ML297 administration improves CFM consolidation in a sleep-dependent manner.** (***A***) Experimental design. Mice underwent single-trial CFC at lights on, were subsequentially administered vehicle or ML297 (30 mg/kg) via i.p. injection and returned to their home cage. Mice in the two drug treatment groups were then allowed *ad lib* sleep or were sleep deprived over the first 6 hr post-CFC, after which all mice were allowed to sleep freely in their home cage until CFM testing the following day at lights on. (***B***) Total amounts of observed sleep during the first 6 hr post-CFC were similar for the two *ad lib* sleep groups. Bars indicate mean ± SEM, *n* = 5 and 6 mice, respectively, for vehicle and ML297. (***C***) Vehicle and ML297 groups in both *ad lib* sleep and sleep deprivation (SD) conditions had similar freezing behavior during training (pre-shock and post-shock). *n* = 5 and 6 mice, respectively, for vehicle and ML297 groups with *ad lib* sleep, and *n* = 7 and 6 mice, respectively, for vehicle and ML297 groups with SD. Freezing behavior during recall, and changes in freezing from the CFC pre-shock period, were significantly greater in freely sleeping ML297-treated mice compared with freely sleeping vehicle-treated mice. SD reduced CFM-associated freezing behavior in both treatment groups, which did not differ from one another. *n* = 5 and 6 mice, respectively, for vehicle and ML297 with *ad lib* sleep, and *n* = 7 and 6 mice, respectively, for vehicle and ML297 with SD. *, **, *** and **** indicate *p* < 0.05, *p* < 0.01, *p* < 0.001, and *p* < 0.0001, respectively, Tukey’s *post hoc* test.

CFM recall was tested for all mice 24 hr after training (Supplementary Figure 5A). The total time spent asleep over the first 6 hr following CFC was similar for the two freely sleeping groups, with no significant effect of ML297 on total sleep time (two-tailed, unpaired *t*-test; *p* = 0.4077; *t*, *df* = 0.8684, 9) (Figure 5B). Mice were video monitored during both CFC and CFM recall testing; representative heat maps showing time-in-location within the CFC chamber during training and testing can be seen in Supplementary Figure S8. As expected, during CFC training, freezing behavior was similar between groups during both pre-shock (two-way ANOVA; *p*(treatment) = 0.9848; *F*(1, 20 = 0.3134); *p*(sleep condition) = 0.0669; *F*(1, 20 = 3.754)) and post-shock (two-way ANOVA; *p*(treatment) = 0.4845; *F*(1, 20 = 0.5075); *p*(sleep condition) = 0.0803; *F*(1, 20 = 3.394)) intervals (Supplementary Figure 5C). 24 hr after CFC, mice were returned to the CFC chamber to test CFM recall. There were significant effects of both prior sleep condition and drug treatment on freezing during recall (two-way ANOVA; *p*(treatment) = 0.00213; *F*(1, 20 = 14.03); *p*(sleep condition) < 0.0001; *F*(1, 20 = 39.71)), and on the change in freezing between recall and training (two-way ANOVA; *p*(treatment) = 0.0022; *F*(1, 20 = 12.29); *p*(sleep condition) < 0.0001; *F*(1, 20 = 31.07), Figure 5C). As expected, freely sleeping vehicle-treated mice had superior CFM comparted to vehicle-treated SD mice (Tukey’s *post hoc* test, *p* = 0.0140) (Figure 5C). Freely sleeping ML297-treated mice showed stronger CFM than all other groups tested (Tukey’s *post hoc* test vs. Sleep+ML297: Sleep+Vehicle, *p* = 0.0423, SD+Vehicle, *p* < 0.0001, and SD+ML297, *p* = 0.0012). However, SD disrupted CFM consolidation regardless of ML297 treatment—i.e. there was no effect of ML297 when mice were sleep deprived (Tukey’s *post hoc* test for vehicle vs. ML297 in the SD condition, *NS*). These findings support the conclusion that sleep is required for ML297-mediated improvements in CFM consolidation. Together, these data suggest that GIRK1/2 channel activation promotes memory consolidation via sleep-dependent mechanisms.

### ML297-mediated improvement in CFM consolidation is associated with greater hippocampal activation during subsequent recall.

**Figure 6.**
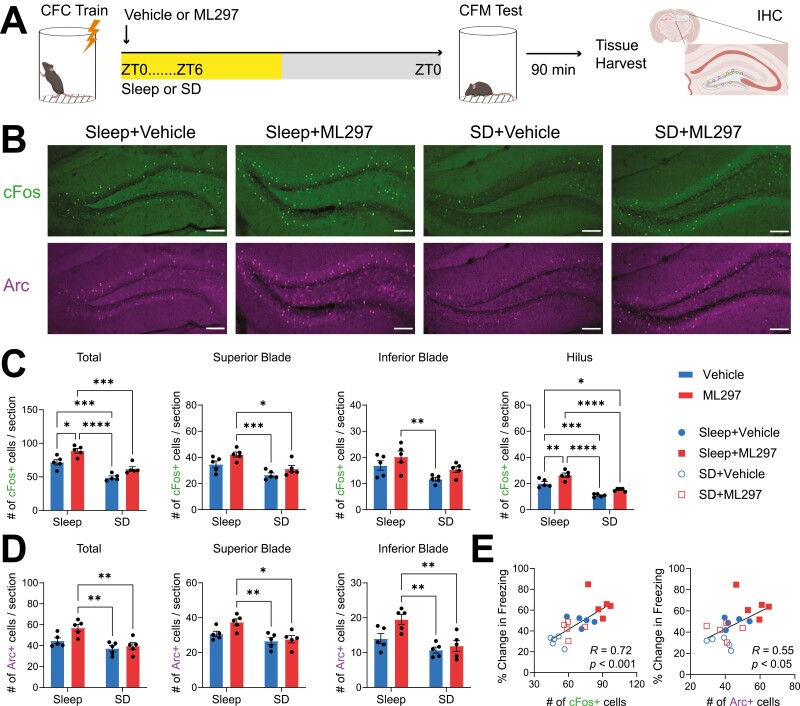
**Post-CFC ML297 increases the number of active neurons in DG during subsequent CFM recall, in a sleep-dependent manner.** (***A***) Experimental paradigm. Mice underwent single-trial CFC, were administered vehicle or ML297 (30 mg/kg) following training, and then were either allowed *ad lib* sleep or underwent 6-hr sleep deprivation (SD). 24 hr after CFC, mice were tested for CFM, and perfused 90 min later to immunohistochemically quantify recall-associated cFos and Arc IEG expression in dorsal hippocampus. *n* = 5 mice/ group. (***B***) Representative images of cFos+ and Arc+ DG neurons following CFM recall in the four treatment groups. Scale bar = 100 µm. (***C***) Mice allowed *ad lib* sleep had significantly increased cFos+ neuron counts across DG compared to both SD groups. ML297 increased cFos+ neuron numbers further in freely sleeping mice. Similar patterns were observed for the two DG granule cell blades, and for the DG hilus. (***D***) Arc+ neuron counts across DG were also reduced in both SD groups. ML297 administration led to a trend for higher overall Arc+ DG neurons relative to vehicles in freely sleeping mice. *, **, ***, and **** indicate *p* < 0.05, *p* < 0.01, *p* < 0.001, and *p* < 0.0001, respectively, Sidak’s *post hoc* test. (***E***) Higher numbers of cFos+ and Arc+ neurons across DG at recall reflected the success of CFM consolidation across individual mice. *R* and *P* values are shown for Pearson correlation.

The major input to hippocampus from the neocortex is relayed through the DG. Acting as a gateway to the rest of the hippocampus, the DG receives sensory and non-sensory information from the rest of the neocortex via entorhinal cortical input. Neuronal immediate early gene (IEG) expression increases among DG granule cells during both initial learning and memory retrieval, and granule cell activation plays a causal role in recall [67]. We tested whether changes in DG activity during CFM recall were associated with sleep- and ML297-mediated improvements in CFM consolidation, by quantifying cFos and Arc expression in hippocampus. After CFM recall, mice were returned to their home cages; 90 min later they were perfused to quantify protein products of IEG expression associated with the recall. We found a significant effects of both sleep and ML297 treatment on cFos expression in the DG (two-way ANOVA; *p*(sleep condition) < 0.0001, *F* (1, 16) = 57.37; *p*(treatment) = 0.0003, *F*(1, 16 = 20.80); Figure 6A–B). ML297-treated mice allowed *ad lib* sleep had significantly increased cFos+ cell counts in DG during recall compared to both SD groups, and freely sleeping vehicle-treated counterparts (Sidak’s *post hoc* test vs. Sleep+ML297: Sleep + Vehicle, *p* = 0.0116; SD + Vehicle, *p* < 0.0001; SD+ML297, *p* = 0.0002; Figure 6B–C). SD also disrupted recall-associated DG cFos expression in vehicle-treated mice (Sidak’s *post hoc* test, *p* = 0.0010; Figure 6C). Similar recall-associated patterns were observed for DG Arc expression (two-way ANOVA; *p*(sleep condition) = 0.0009; *F*(1, 16 = 16.49); *p*(treatment) = 0.0304; *F*(1, 16 = 5.641)) (Figure 6B, D), with reduced numbers of Arc + neurons in SD mice (Sidak’s *post hoc* test vs. Sleep + ML297: SD + Vehicle, *p* = 0.0020; SD+ML297, *p* = 0.0057) (Figure 6D). Overall expression of both IEGs in DG at recall was predictive of successful recall, with higher numbers of cFos+ and Arc+ neurons corresponding to increased freezing behavior during CFM testing (cFos: Pearson correlation coefficient, *R* = 0.7225, *p* = 0.0003; Arc: Pearson correlation coefficient, *R* = 0.5521, *p* = 0.0116) (Figure 6E).

We also quantified IEG expression within individual subregions of the DG to examine whether changes associated with recall in the four treatment groups were region-specific. As shown in Figure 6C–D, similar patterns of expression were observed in the granule cell body layer of both the superior and inferior blade of DG, and in the DG hilus. For cFos expression, we found significant effect of both sleep and treatment in both the superior and inferior blades (superior: two-way ANOVA; *p*(sleep condition) = 0.0005, *F* (1, 16) = 19.15; *p*(treatment) = 0.0127 *F*(1, 16 = 7.863); inferior: two-way ANOVA; *p*(sleep condition) = 0.0051, *F* (1, 16) = 10.53; *p*(treatment) = 0.0379; *F*(1, 16 = 5.123)), as well as in the hilus (two-way ANOVA; *p*(sleep condition) < 0.0001, *F* (1, 16) = 82.24; *p*(treatment) = 0.0002; *F*(1, 16 = 22.19); Figure 6C). Similarly, Arc expression patterns in superior and inferior blades after recall were similar to overall Arc+ neuron numbers (superior: two-way ANOVA; *p*(sleep condition) = 0.0021, *F* (1, 16) = 13.37; *p*(treatment) = 0.0584 *F*(1, 16 = 4.155); inferior: two-way ANOVA; *p*(sleep condition) = 0.0010, *F* (1, 16) = 16.03; *p*(treatment) = 0.0246; *F*(1, 16 = 6.154); Figure 6D).

**Figure 7.**
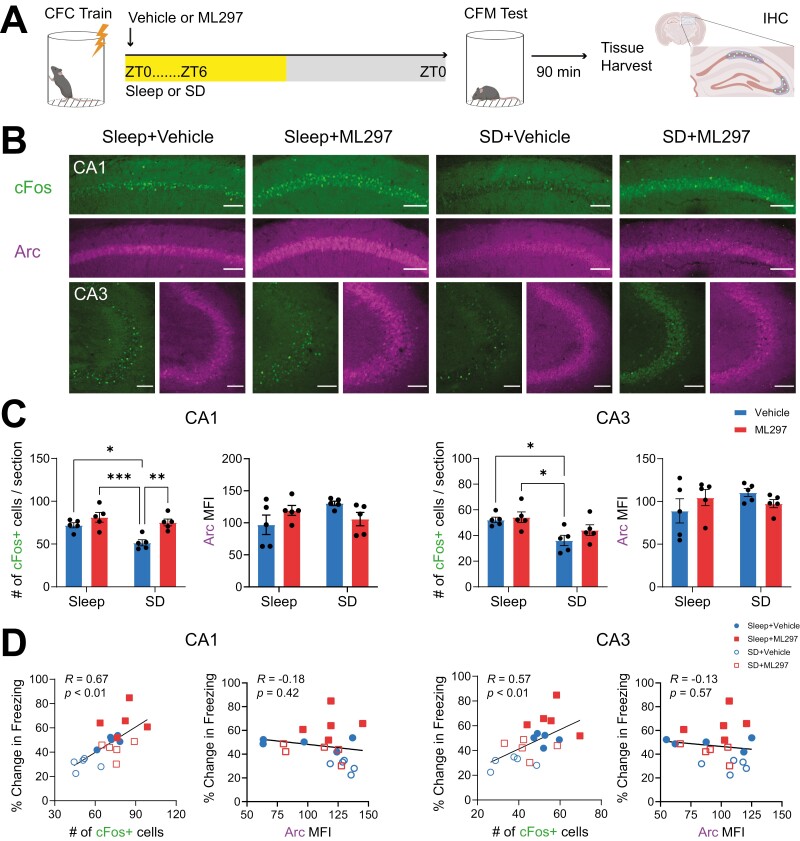
**Post-CFC ML297 increases the number of active neurons in CA1 during subsequent CFM recall, in a sleep-independent manner.** (***A***) Experimental design, as in Figure 6A. (***B***) Representative images of cFos+ neurons and Arc+ mean fluorescence intensity (MFI) in the pyramidal layers in CA1 and CA3 following CFM recall in the four groups. Scale bar = 100 µm. (***C***) Post-CFC sleep deprivation (SD) significantly decreased the number of cFos+ neurons in CA1 and CA3 during recall in vehicle-treated mice. In CA1 only, ML297 increased cFos+ neuron numbers after recall in SD mice as well as freely sleeping mice. No significant changes in Arc MFI were observed with SD or drug treatment in either CA1 or CA3. *, **, and *** indicate *p* < 0.05, *p* < 0.01, and *p* < 0.001, respectively, Sidak’s *post hoc* test. (***D***) Numbers of cFos+ neurons in both CA1 and CA3 during recall correlated with CFM recall performance. No correlations were observed between freezing behavior and Arc MFI expression. *R* and *P* values are shown for Pearson correlation.

To better understand how recall-associated neuronal activation is affected across the rest of the hippocampal circuit as a function of post-learning sleep and ML297, we also examined IEG expression within the pyramidal cell layers of CA1 and CA3 after CFM recall (Figure 7A–B). Recall-driven cFos+ neuron numbers in CA1 varied significantly as a function of prior sleep and drug treatment in CA1 (CA1: two-way ANOVA; *p*(sleep condition) = 0.0060, *F* (1, 16) = 10.03; *p*(treatment) = 0.0011 *F*(1, 16 = 15.72); Figure 7C). Critically, however, cFos+ neuron numbers in CA1 were increased by ML297, even in SD mice (Sidak’s *post hoc* test vs. SD + Vehicle, *p* = 0.0058). This suggests that CA1 cFos+ cell numbers are enhanced by ML297 administration even in a scenario where consolidation of CFM has been disrupted by SD. Nonetheless, higher numbers of cFos+ neurons in CA1 were associated with better CFM recall (i.e. higher levels of freezing; Pearson correlation coefficient, *R* = 0.6701, *p* = 0.0012; Figure 7D). cFos+ neuron numbers in CA3 showed a similar overall pattern, but varied significantly as a function of sleep only (two-way ANOVA; *p*(sleep condition) = 0.0030, *F* (1, 16) = 12.25; *p*(treatment) = 0.1914; *F*(1, 16 = 1.861); Figure 7C). cFos+ cell counts in CA3 also reflected freezing levels during recall for individual animals (Pearson correlation coefficient, *R* = 0.5719, *p* = 0.0084; Figure 7D).

Due to the widespread nature of Arc expression in CA1 and CA3, we quantified the mean fluorescence intensity (MFI) of Arc immunostaining in these sections after recall, using previously described methods [12]. Using this strategy for quantification of Arc, no significant differences were observed across groups for any of the groups in either CA1 or CA3 (Figure 7C), and MFI values were not predictive of freezing behavior during recall (Figure 7D).

Taken together, these studies suggest that both post-CFC sleep (vs. SD), and post-CFC administration of ML297, can increase dorsal hippocampus neuronal activation during subsequent CFM recall. These effects on hippocampal activation (particularly on neuronal activation in DG) during recall mirror, and are positively correlated with, freezing behavior during recall. These findings also support the idea that GIRK1/2 activation alters hippocampal network-level processes involved in consolidation in a sleep-dependent manner, leading to sleep-dependent changes in activation during recall.

## Discussion

We find that in the hours immediately following CFC, direct GIRK1/2 channel activation can increase REM sleep, restoring REM sleep architecture (normally suppressed after fear learning) to baseline levels, and improving sleep-dependent consolidation of fear memory. We find that ML297-associated increases in overall REM sleep amounts, bout durations, and bout numbers (and reduced latency to REM sleep) co-occur with increased NREM spindle power over the first 6 r post-CFC (and higher spindle power continuing into the subsequent dark phase). It is likely that these changes are driven by the same underlying mechanism, due to the increasingly well-established causal relationship between spindle-rich NREM sleep and transitions from NREM into REM sleep [65, 66]. In other words, it is likely that transitions into REM sleep after ML297 administration reflect the normal physiology of such transitions. Critically, all of the NREM and REM sleep changes caused by ML297 in the hours following CFC appear to be renormalizing sleep architecture to what is typically observed under baseline conditions (i.e., in the absence of fear learning). Two features of these findings are worth noting. First, the ML297-induced changes in sleep architecture that are correlated with successful CFM consolidation occur almost exclusively within a window of time (i.e. the first 6 hr after CFC) where SD is sufficient to disrupt the consolidation process [6, 16–18]. Second, post-CFC SD is sufficient to prevent the CFM consolidation benefits of ML297 administration.

It is worth noting that both spindle-rich NREM sleep (such as that present at the transition to REM) and REM sleep have been linked to memory storage, across species - from humans to rodent models [1, 68, 69]. NREM spindles have received a great deal of recent study due to their linkage to sleep-related improvements on a range of mnemonic tasks, and to sleep-dependent synaptic plasticity in neocortex [3, 21, 33, 55, 70, 71]. Within the hippocampus, spindles and other NREM-associated electrophysiological [19, 21] and neuromodulatory [11, 22] changes have been mechanistically linked to successful CFM consolidation. Our present data suggest that REM is at least equally vital for CFM, and support a growing body of data indicating that REM-specific features of post-learning hippocampal activity [18, 28] and gene expression [72–74] are essential for the consolidation process. Together, our findings support the notion that post-CFC REM sleep plays a causal role in promoting fear memory consolidation.

To better understand the link between our behavioral results and hippocampal network-level events underlying successful memory consolidation, we examined IEG expression within the hippocampus following CFM recall. We find that just as recall itself is suppressed after post-CFC SD, the number of cFos+ and Arc+ neurons in DG after recall, as well as the number of cFos+ neurons in downstream regions CA3 and CA1, is decreased in SD mice. This is consistent with the idea that hippocampal activation during recall is reduced overall after SD-disrupted consolidation. Because mice are given an adequate opportunity for recovery sleep between SD and recall (i.e. 18 hr from ZT6 to ZT0 the following day), we believe that this alteration is due to a long-term change in the strength of the memory trace itself, rather than an acute effect of SD on hippocampal activation [11, 12, 75]. In other words, an increase in the number of neurons active at recalling following *ad lib* sleep would reflect more neurons’ inclusion into the hippocampal “engram”, while decreases after SD would reflect a reduction in neuronal incorporation into the memory trace. Intriguingly, ML297 administration after CFC in freely sleeping mice results in a further increase in the number of IEG+ neurons in DG, where recall-activated neurons are generally sparser, but does not affect IEG+ numbers when administered in the context of SD. These sleep-dependent effects of ML297 on DG neurons’ activity during recall closely reflect effects of ML297 on CFM consolidation. In contrast, in CA1, ML297 increases the number of recall-activated cFos+ neurons, regardless of whether mice are freely sleeping or sleep-deprived. This suggests additional, sleep-independent effects of ML297 within CA1, the region of hippocampus where GIRK1/2 channels are most abundant [44, 48, 52]. Overall, our data suggest that GIRK channel activation has sleep-dependent and sleep-independent effects on the dorsal hippocampal network in the context of consolidation, leading to the incorporation of more neurons into the CFM engram, which is evident in the pattern of network activation during CFM recall.

While numerous genetic findings suggest that loss of GIRK channel activity disrupts hippocampal memory processing [76], their precise molecular role in this process remains unclear. In vitro studies have shown that in the hippocampus (e.g. CA1) GIRK channel activation induces hyperpolarization, reduces neuronal excitability, and suppresses LTP [49, 50, 53]. However, it is unknown how these effects translate to in vivo function, and particularly how these changes are modulated in different brain states (such as wake vs. NREM and REM sleep). Future studies will be needed to disentangle the relationship between the direct cellular effects of ML297 administration, its behavioral effects (e.g. sleep-promoting, anxiolytic), and its mnemonic effects during the memory consolidation process.

It is also worth noting that GIRK1/2 is expressed in other brain regions, including the neocortex and the thalamus [77]. The effects of ML297 treatment on delta and spindle oscillations may very well be mediated by activation of GIRK1/2 in these structures [3, 4]. While our present findings are focused on drug effects on subsequent recall-associated activation in the hippocampus, it is very plausible that ML297 also affects other structures important for CFM, including thalamocortical circuits and the amygdala.

Recent data have implicated GIRK channels as a target for therapeutics in various neurological and psychiatric conditions including epilepsy, Alzheimer’s disease, substance abuse, and anxiety disorders [52, 76]. Our present data support the recent suggestion [40] that GIRK1/2 activation via ML297 could also be beneficial as a hypnotic. Beyond this, our data demonstrate that this hypnotic agent restores physiological REM sleep (whose disruption by fear learning is well-established [23]) to promote sleep-dependent memory consolidation. These findings have important ramifications for the treatment of disorders—including neurodevelopmental disorders, dementia, and anxiety disorders—where both sleep architecture and cognitive function are disrupted.

## Supplementary Material

zsac301_suppl_Supplementary_Material

## Data Availability

Data and MATLAB code used for EEG/EMG analysis in these studies will be made available upon reasonable request.
