# Supplementary material for: Atypical hypnotic compound ML297 restores sleep architecture immediately following emotionally valenced learning, to promote memory consolidation and hippocampal network activation during recall
Source: Sleep. 2022 Dec 13;46(3):zsac301. doi: 10.1093/sleep/zsac301 (PMC9995787; doi:10.1093/sleep/zsac301)
Supplement: zsac301_suppl_Supplementary_Material [file zsac301_suppl_supplementary_material.pdf]

Supplementary Material for:

Atypical hypnotic compound ML297 restores sleep architecture immediately following emotionally-valenced learning, to promote memory consolidation and hippocampal network activation during recall

Jessy D. Martinez[1], William P. Brancaleone[2], Kathryn G. Peterson[2], Lydia G. Wilson[1], Sara J. Aton[1#]

[1] Department of Molecular, Cellular, and Developmental Biology, University of Michigan, Ann Arbor, MI, USA 48109

[2] Undergraduate Program in Neuroscience, University of Michigan, Ann Arbor, MI, USA 48109

[#] **Corresponding Author**

Dr. Sara J. Aton
University of Michigan
Department of Molecular, Cellular, and Developmental Biology
4268 Biological Sciences Building
1105 N. University Ave
Ann Arbor, MI, USA 48109
Phone: (734) 615-1576
Email: saton@umich.edu

<u>Supplementary Figure Legends:</u>

<u>Figure S1:</u> Representative heat maps of time-in-location for individual EEG-implanted mice during CFC training and CFM testing (***A***) Heat maps indicating time spent in various locations within the conditioning chamber, for representative vehicle- and ML297-treated mice during CFC and CFM testing. Red represents locations in the CFC chamber where mice spent the most time, while blue represents locations in the CFC chamber where mice spent the least time.

<u>Figure S2:</u> NREM and REM sleep architecture are similar during the dark phase (ZT12-24) during baseline recording and following CFC. (***A***) NREM and (***B***) REM total sleep behavior during baseline across 6-h periods of dark phase were similar for vehicle and ML297 groups. (***C***) NREM and (***D***) REM total sleep behavior post-CFC during the dark phase were similar for vehicle and ML297 groups. n = 6 mice/group

<u>Figure S3:</u> Wake architecture across the light:dark cycle. Recording time percentage spent in wake across (***A***) baseline and (***B***) post-CFC periods were generally similar for vehicle and ML297 groups. When percent time in wake was calculated across the first 6 h of the dark phase (ZT12-18), vehicle mice spent more time in wake compared to ML297 mice (* indicates $p < 0.05$, Sidak's *post hoc* test vs. vehicle n = 6 mice/group). Gray shaded areas represent lights off.

<u>Figure S4:</u> Wake architecture in vehicle and ML297 groups. Mean bout duration for wake during (***A***) baseline and (***B***) post-CFC periods in vehicle and ML297 groups were similar. Number of wake bouts during (***C***) baseline and (***D***) post-CFC periods in vehicle- and ML297-treated mice were also similar. Gray shaded areas represent lights off. n = 6 mice/group

<u>Figure S5:</u> NREM and REM EEG spectral power across the dark phase (ZT12-24). EEG power spectra (recorded over visual cortex, bilaterally) for the dark cycle (12-24) are shown for vehicle and ML297 groups during NREM baseline (***A***) and post-CFC (***B***), and during REM baseline (***C***) and post-CFC (***D***). Values indicate % of total spectral power at each frequency band, mean ± SEM; n = 6 mice/group. No significant changes between vehicle and ML297 groups were observed during baseline or post-CFC. n = 6 mice/group. (***E-F***) Change in spectral power from baseline for NREM and REM sleep. No changes in spectral power from baseline were observed at specific delta or theta frequencies.

<u>Figure S6:</u> NREM sleep spindle features in the dark phase (ZT12-24) following CFC. NREM spindle density (***A***) and spindle duration (***B***) were similar between vehicle and ML297 groups for hours 12-24 during baseline. (***C***) NREM EEG spectral power within the spindle/sigma frequency band (7-15 Hz) was similar between groups at baseline. NREM spindle density (***D***) and mean duration (***E***) were similar between both vehicle- and ML297-treated mice following CFC at ZT12-24. (***F***) Average NREM spindle power was significantly different in vehicle-treated mice relative to ML297-treated mice, over the entire dark phase following CFC (two-way RM ANOVA; p(time of day x treatment) = 0.0300; F(5, 50 = 2.718). Values indicate mean ± SEM; n = 6 mice/group.

<u>Figure S7:</u> Validation of visual observation-based sleep scoring and SD methods. (***A***) Total percentage of time spent in sleep, compared for the same cohort of mice using EEG/EMG and visual scoring (VS) over a 6-h period (ZT0-6), was similar. Values indicate mean ± SEM; n = 4 mice/group. (***B***) Sleep architecture for EEG recorded mice. (***C***) Total sleep across 2-h bins for EEG vs. VS methods. (***D***) Total percentage of recording time spent in wake and NREM sleep in EEG/EMG recorded mice across 6 h SD by gentle handling. As a group, SD mice spent 90% of the SD period awake, and did not enter REM sleep. (***E***) Across SD, an increasing number of experimental interventions (cage tapping, cage shaking, nest disturbances) were required to

maintain wakefulness, consistent with increasing sleep pressure. (***F***) Spindle density during brief periods of NREM within SD was similar to that observed during *ad lib* sleep.

Figure S8: Representative heat maps of time-in-location for individual non-implanted mice during CFC training and CFM testing. (***A***) Heat maps indicating time spent in various locations within the CFC chamber for representative vehicle and ML297 mice during CFC and CFM testing. Red represents locations in the CFC chamber where mice spent the most time, while blue represents locations in the CFC chamber where mice spent the least time.

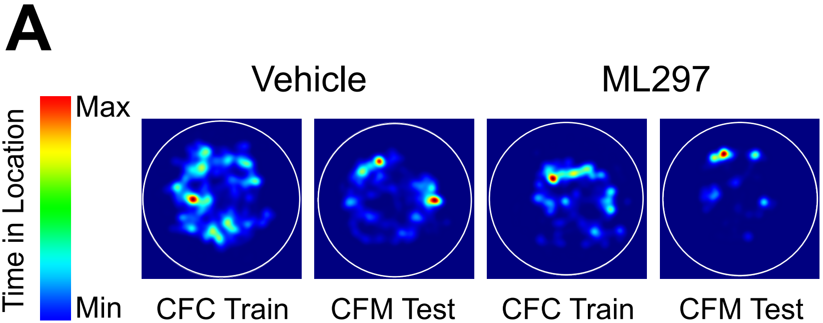
A
Vehicle
ML297
Time in Location
Max
Min
CFC Train
CFM Test
CFC Train
CFM Test

Figure S1

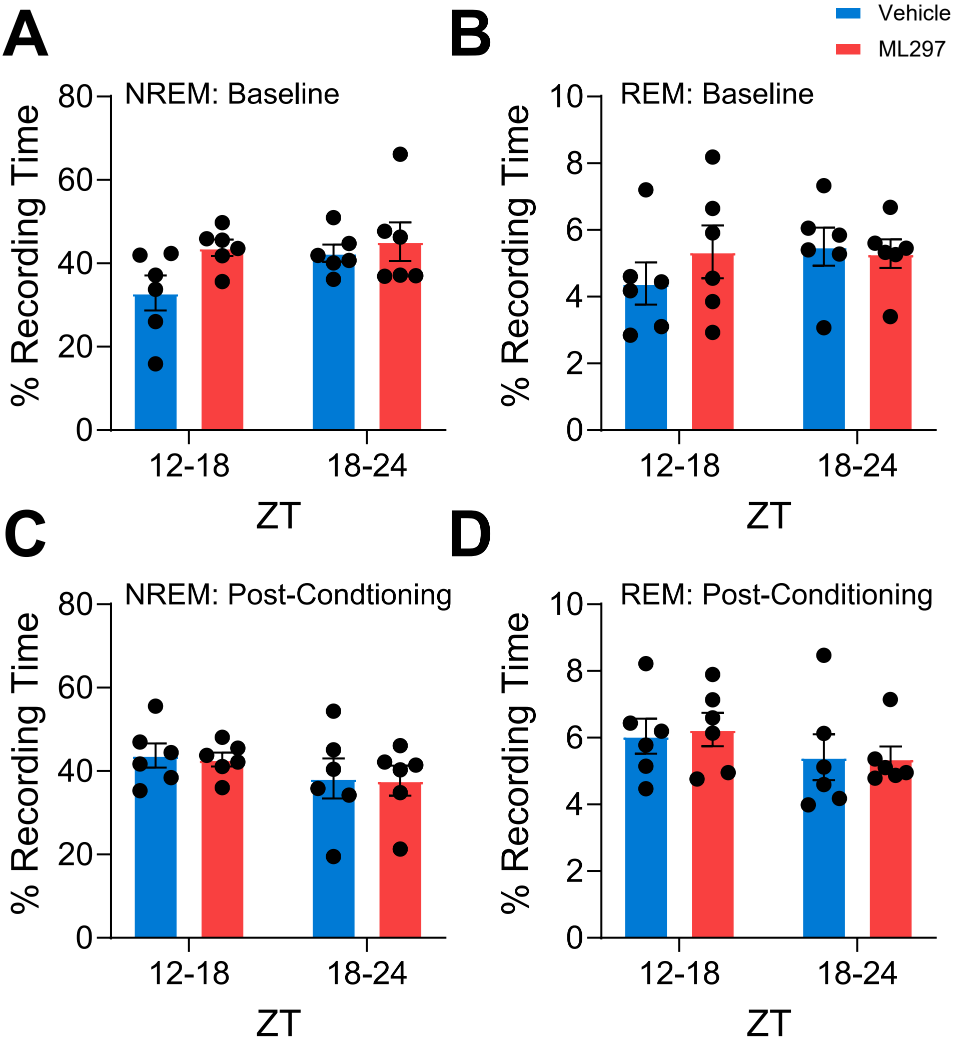
Vehicle
ML297
A
NREM: Baseline
% Recording Time
80
60
40
20
0
12-18
18-24
ZT
B
REM: Baseline
% Recording Time
10
8
6
4
2
0
12-18
18-24
ZT
C
NREM: Post-Condtioning
% Recording Time
80
60
40
20
0
12-18
18-24
ZT
D
REM: Post-Conditioning
% Recording Time
10
8
6
4
2
0
12-18
18-24
ZT

Figure S2

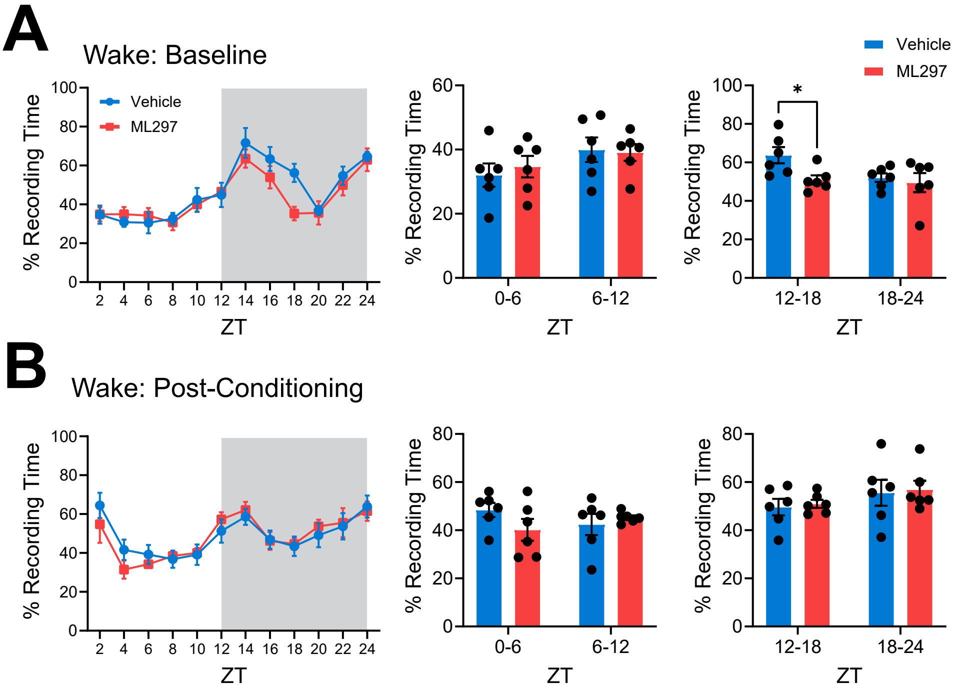
A
Wake: Baseline
% Recording Time
Vehicle
ML297
ZT
0-6
6-12
12-18
18-24
*
B
Wake: Post-Conditioning

Figure S3

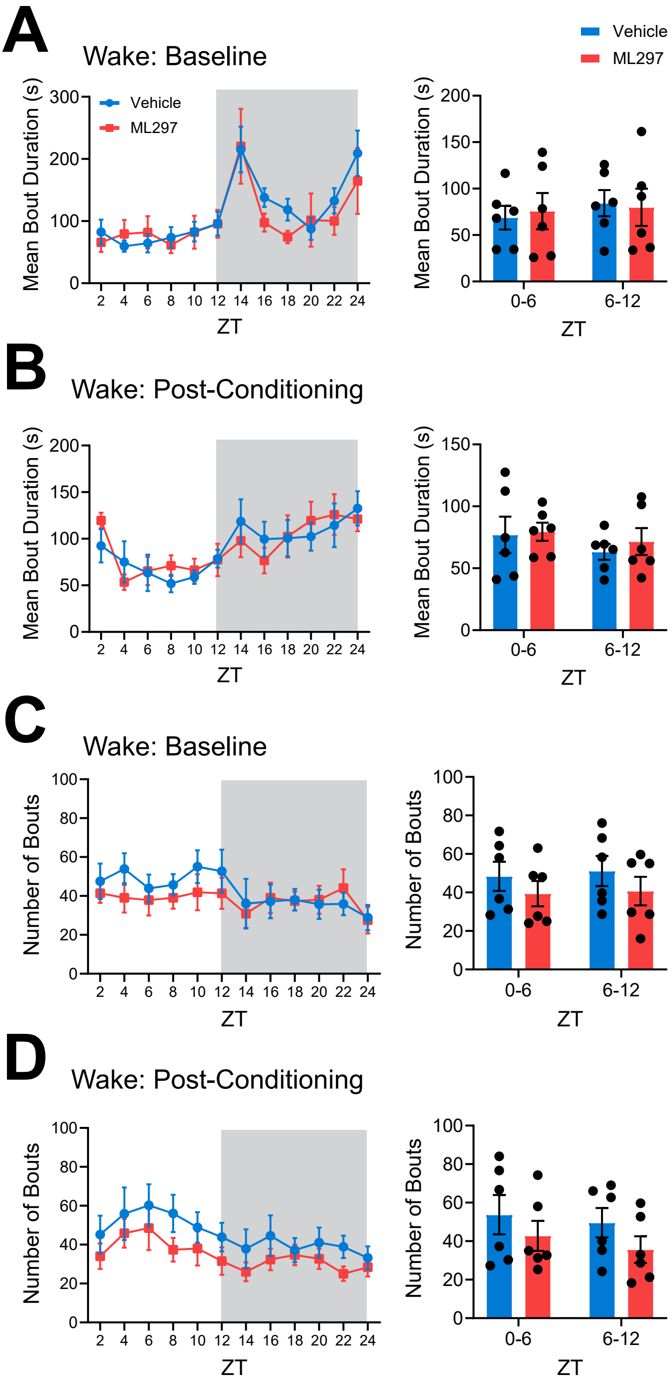
A
Wake: Baseline
Vehicle
ML297
Mean Bout Duration (s)
ZT
0-6
6-12
B
Wake: Post-Conditioning
Mean Bout Duration (s)
ZT
0-6
6-12
C
Wake: Baseline
Number of Bouts
ZT
0-6
6-12
D
Wake: Post-Conditioning
Number of Bouts
ZT
0-6
6-12

Figure S4

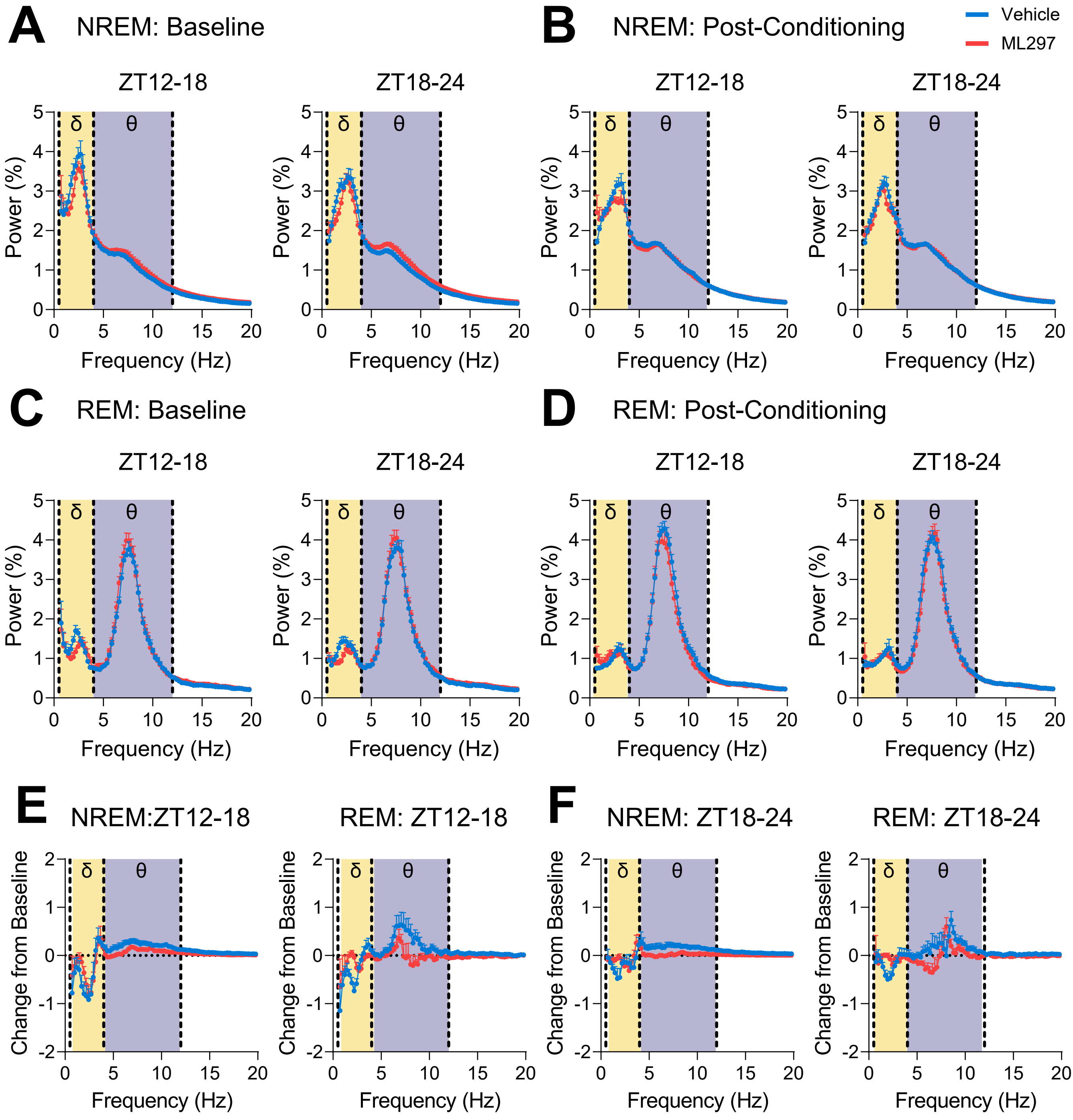
A
NREM: Baseline
B
NREM: Post-Conditioning
Vehicle
ML297
ZT12-18
ZT18-24
δ
θ
Power (%)
Frequency (Hz)
C
REM: Baseline
D
REM: Post-Conditioning
E
NREM:ZT12-18
REM: ZT12-18
F
NREM: ZT18-24
REM: ZT18-24
Change from Baseline

Figure S5

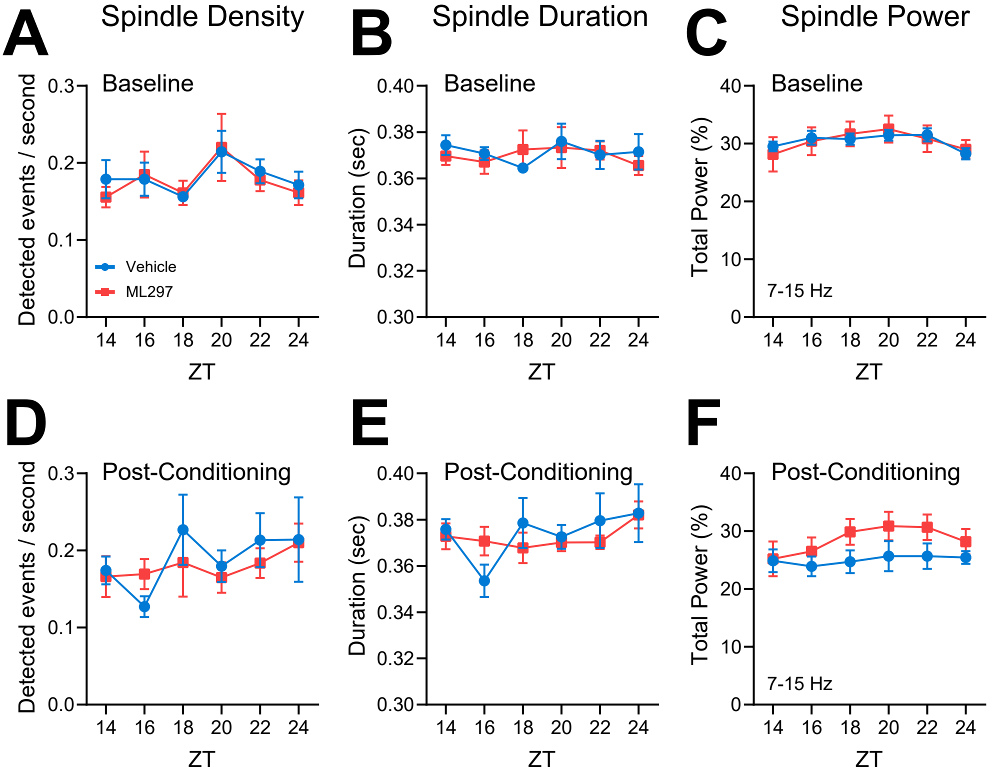
A
Spindle Density
Baseline
Detected events / second
0.0
0.1
0.2
0.3
Vehicle
ML297
14
16
18
20
22
24
ZT
B
Spindle Duration
Baseline
Duration (sec)
0.30
0.32
0.34
0.36
0.38
0.40
C
Spindle Power
Baseline
Total Power (%)
0
10
20
30
40
7-15 Hz
D
Post-Conditioning
E
Post-Conditioning
F
Post-Conditioning
7-15 Hz

Figure S6

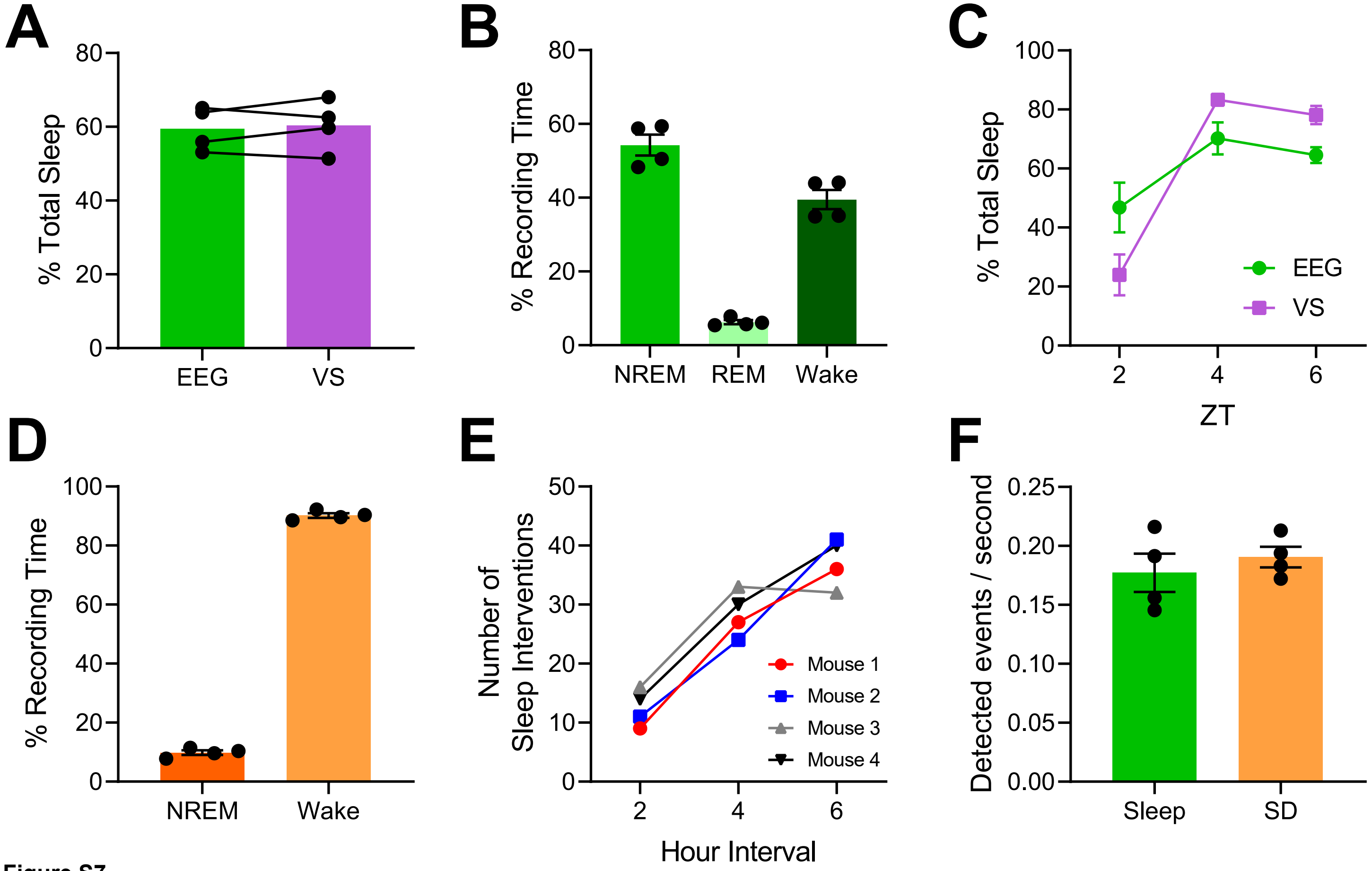
A
80
60
40
20
0
% Total Sleep
EEG
VS
B
80
60
40
20
0
% Recording Time
NREM
REM
Wake
C
100
80
60
40
20
0
% Total Sleep
EEG
VS
2
4
6
ZT
D
100
80
60
40
20
0
% Recording Time
NREM
Wake
E
50
40
30
20
10
0
Number of
Sleep Interventions
Mouse 1
Mouse 2
Mouse 3
Mouse 4
2
4
6
Hour Interval
F
0.25
0.20
0.15
0.10
0.05
0.00
Detected events / second
Sleep
SD

Figure S7

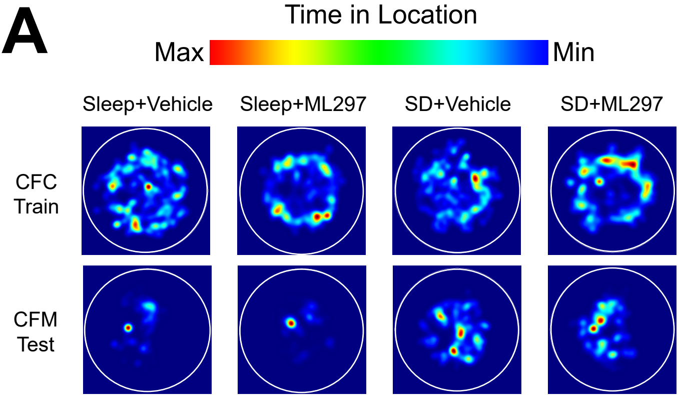
A
Time in Location
Max
Min
Sleep+Vehicle
Sleep+ML297
SD+Vehicle
SD+ML297
CFC
Train
CFM
Test

Figure S8
